# PINK1-induced mitophagy promotes neuroprotection in Huntington's disease

**DOI:** 10.1038/cddis.2014.581

**Published:** 2015-01-22

**Authors:** B Khalil, N El Fissi, A Aouane, M-J Cabirol-Pol, T Rival, J-C Liévens

**Affiliations:** 1 Aix-Marseille Université, CNRS, Centre de Recherche en Neurobiologie et Neurophysiologie de Marseille, UMR 7286, 13344 Marseille, France; 2 Aix-Marseille Université, CNRS, Institut de Biologie du Développement de Marseille, UMR 7288, 13288 Marseille, France

## Abstract

Huntington's disease (HD) is a fatal neurodegenerative disorder caused by aberrant expansion of CAG repeat in the *huntingtin* gene. Mutant Huntingtin (mHtt) alters multiple cellular processes, leading to neuronal dysfunction and death. Among those alterations, impaired mitochondrial metabolism seems to have a major role in HD pathogenesis. In this study, we used the *Drosophila* model system to further investigate the role of mitochondrial damages in HD. We first analyzed the impact of mHtt on mitochondrial morphology, and surprisingly, we revealed the formation of abnormal ring-shaped mitochondria in photoreceptor neurons. Because such mitochondrial spheroids were previously detected in cells where mitophagy is blocked, we analyzed the effect of PTEN-induced putative kinase 1 (PINK1), which controls Parkin-mediated mitophagy. Consistently, we found that PINK1 overexpression alleviated mitochondrial spheroid formation in HD flies. More importantly, PINK1 ameliorated ATP levels, neuronal integrity and adult fly survival, demonstrating that PINK1 counteracts the neurotoxicity of mHtt. This neuroprotection was Parkin-dependent and required mitochondrial outer membrane proteins, mitofusin and the voltage-dependent anion channel. Consistent with our observations in flies, we demonstrated that the removal of defective mitochondria was impaired in HD striatal cells derived from HdhQ111 knock-in mice, and that overexpressing PINK1 in these cells partially restored mitophagy. The presence of mHtt did not affect Parkin-mediated mitochondrial ubiquitination but decreased the targeting of mitochondria to autophagosomes. Altogether, our findings suggest that mitophagy is altered in the presence of mHtt and that increasing PINK1/Parkin mitochondrial quality control pathway may improve mitochondrial integrity and neuroprotection in HD.

Huntington's pathology is a devastating inherited neurodegenerative disease clinically characterized by involuntary movements, cognitive decline, emotional changes and dementia. Huntington's disease (HD) results from an increased length of a polyglutamine stretch in the N terminus of Huntingtin protein, due to expanded CAG repeat in the first exon of the *IT15/huntingtin* gene. Symptoms are observed when patients carry more than 36 CAG repeats and there is still no cure to halt the progression, which leads to death after 10 to 20 years. Despite ubiquitous expression of mutant Huntingtin (mHtt) throughout the brain, only specific neuronal sub-populations degenerate mostly in the striatum and, to a lesser extent, in the cerebral cortex.

The presence of mHtt leads to multiple cellular dysfunctions, including alterations in intracellular signaling pathways, defects in cellular trafficking, transcription deregulation, abnormal synaptic transmission, proteasomal dysfunction and mitochondrial alterations. One challenging question is to determine to what extent each of those defects contributes to the disease progression.

Growing evidence suggest that mHtt disrupts mitochondrial functions, resulting in energetic defect, reactive oxygen species overload and release of proapoptotic molecules. More specifically, the respiratory chain is affected with impairment of the mitochondrial complex II/III activity in the caudate and putamen samples of HD patients.^1, 2^ The maintenance of functional mitochondria requires biogenesis as well as mitochondrial fusion/fission dynamics to replenish stores of damaged components. Peroxisome proliferator-activated receptor gamma coactivator-1-alpha (PGC-1*α*) is a key transcriptional coactivator that controls mitochondrial biogenesis and energy metabolism. PGC-1*α* is downregulated by mHtt through interference with the CREB/TAF4-dependent transcriptional pathway.^3^ More recently, fragmented mitochondria were linked to HD,^4^ due to increased dynamin-related protein 1 (DRP1) activity.^5, 6^ The accumulation of mitochondrial damages in postmitotic neurons is therefore considered as a key process in HD pathogenesis.

The ultimate mechanism that cells use when they are overwhelmed by dysfunctional mitochondria is to remove the damaged organelles by selective autophagy, the so-called mitophagy. PTEN-induced putative kinase 1 (PINK1) is a molecular sensor that accumulates at the outer membrane of damaged mitochondria to recruit the E3 ubiquitin ligase Parkin and initiates the mitophagic process.^7, 8, 9^ So far, the impact of PINK1 and Parkin has been investigated mainly in Parkinson's disease (PD). Indeed, mutations in both genes are directly linked to familial parkinsonism.

In the present study, we first analyzed the impact of mHtt on mitochondrial morphology in a *Drosophila* model of HD. Surprisingly, we revealed the formation of mitochondrial spheroids in non-apoptotic photoreceptor neurons. Because those atypical mitochondria were recently described in cells where mitophagy was blocked,^10, 11^ it suggests that mHtt may cause an imbalance between the accumulation of damaged mitochondria and their clearance through mitophagy. To test this hypothesis, we analyzed the effect of PINK1 overexpression in HD flies. We found that PINK1 reduces mitochondrial spheroid formation and, more importantly, confers substantial protection against mHtt-induced neuronal phenotype in adult flies. We further showed that mHtt disrupted colocalization between mitochondria and autophagosomes and, thereby, the elimination of defective mitochondria in striatal cells derived from knock-in HdhQ111 mice. Consistent with our obsevations made in HD flies, PINK1 overexpression partially restored mitophagy in HdhQ111 cells. Altogether, our results highlight the value of PINK1/Parkin pathway to alleviate mitochondrial defects in HD.

## Results

### Mitochondrial accumulation and spheroid formation precede mHtt-induced neurodegeneration and are suppressed by PINK1

**Figure 1:**
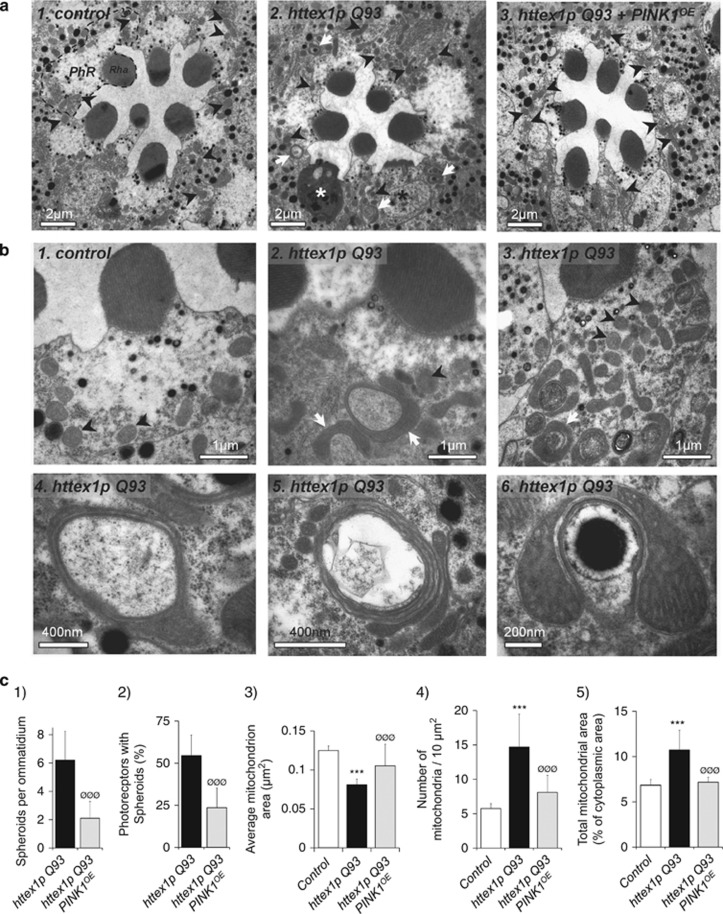
Mitochondrial spheroid formation precedes Httex1p Q93-induced neurodegeneration and is suppressed by *Drosophila* PINK1 overexpression. (**a**) Transmission electron microscopy (TEM) pictures of eye sections from 1-day-old adult *Drosophila*. Panel 1: Ommatidia of control flies normally contain seven visible photoreceptor neurons (PhRs), each photoreceptor harboring a rhabdomere membrane subdomain (Rha) that detects light. Black arrowheads show typical round-shaped mitochondria. Panel 2: Targeted neuronal expression of *httex1p Q93* triggers rhabdomere degeneration (black asterisk) and photoreceptor apoptosis (white asterisk). Circular electron-dense structures corresponding to mitochondrial spheroids (white arrows) are observed in the cytoplasm of non-apoptotic PhRs. Black arrowheads point to round-shaped mitochondria. Panel 3: TEM picture of an ommatidium from 1-day-old *Drosophila* coexpressing *httex1p Q93* and *Drosophila PINK1* (*PINK1*^*OE*^) in neurons. No mitochondrial spheroid is detected in this ommatidium. Black arrows correspond to round-shaped mitochondria. (**b**) High magnification TEM images show that circular electron-dense structures observed in Httex1p Q93 photoreceptors are mitochondrial spheroids. Panels 1 and 2, respectively, show control and Httex1p Q93-expressing photoreceptors with no sign of degeneration. Black arrowheads point to mitochondria with normal round-shaped morphology, whereas white arrows correspond to spheroid mitochondria. Panel 3 represents an *httex1p Q93*-expressing photoreceptor containing a massive accumulation of mitochondria within its cytoplasm (white arrows: mitochondrial spheroids; black arrows: round-shaped mitochondria). Panels 4–6 show the ultrastructure of representative mitochondrial spheroids from *httex1p Q93*-expressing photoreceptors. Mitochondrial spheroids often form ring-like structures harboring an enlarged region. Mitochondrial spheroids can also appear as bended barbell-like structures (panels 5 and 6) sometimes involving multiple mitochondria (panel 5) or even enwrapping cytoplasmic components such as pigment granules (panel 6). (**c**) Quantitative analyses from 1-day-old flies expressing no transgene (control), *httex1p Q93* alone or together with *PINK1* (*PINK1*^*OE*^) in neurons. Histograms show the number of spheroids per ommatidium (histogram 1), the number of photoreceptors containing mitochondrial spheroids (histogram 2), the average area of individual mitochondria (*μ*m^2^) (histogram 3), the number of mitochondria per 10 *μ*m^2^ of the cytoplasm (histogram 4) and the total area occupied by the mitochondria within the photoreceptor cytoplasm (ratio of mitochondrial area/cytoplasmic area in %) (histogram 5). Measurements in histograms 3–5 do not include spheroids. Observations were made on eye sections from at least three independent flies. Spheroid quantifications were performed on 20 ommatidia and 120 non-apoptotic photoreceptors for *httex1p Q93*, and 32 ommatidia and 190 non-apoptotic photoreceptors for *httex1p Q93+PINK1*^*OE*^. Quantification of mitochondrial area, number and density were performed on 51 photoreceptors and 278 mitochondria for control, 55 photoreceptors and 875 mitochondria for *httex1p Q93*, and 58 photoreceptors and 374 mitochondria for *httex1p Q93+PINK1*^*OE*^. Statistics were performed by using the Mann–Whitney *U*-test (***P<0.0001 *versus* control; ^ØØØ^*P*<0.0001 *versus* *httex1p Q93*)

To determine whether neuronal expression of mHtt (Httex1p Q93) in *Drosophila* alters mitochondrial morphology, we performed transmission electron microscopy analysis of the retina, a CNS structure that shows widespread neuronal loss in HD flies.^12^ We analyzed 1-day-old flies expressing mHtt specifically in neurons because the neurodegeneration is moderate at this age, although some photoreceptors start to display rhabdomere atrophy (Figure 1a, panel 2, black asterisk) or cytoplasmic condensation typical of apoptosis (Figure 1a, panel 2, white asterisk). We found that non-apoptotic photoreceptors contained two types of mitochondria: typical round-shaped mitochondria and, unexpectedly, mitochondrial tubules that seem to bend and often fuse at their extremities to adopt a ring-shaped structure (Figure 1b, panels 2–5). Sometimes those bended mitochondria engulf cytoplasmic components such as pigment granules (Figure 1b, panel 6). Those abnormal mitochondria were strikingly reminiscent to the mitochondrial spheroids recently described in cells where mitophagy was blocked.^10, 11^ With the aim of boosting mitophagy, we decided to overexpress PINK1. *Drosophila* PINK1 overexpression (*PINK1*^*OE*^*line*) significantly reduced mitochondrial spheroid formation in photoreceptor neurons (Figure 1a, panel 3 and Figure 1c, histograms 1 and 2). We also performed quantitative analyses on the mitochondria that were not forming spheroids. In photoreceptors, these organelles were generally round shaped, or sometimes appeared as short tubules. *Drosophila* expressing mHtt showed a decrease in the average size (Figure 1c, histogram 3) and an increase in the average number of non-spheroid mitochondria (Figure 1c, histogram 4), suggesting that mHtt leads to mitochondrial fragmentation. More importantly, the total area covered by the mitochondria in the cytoplasm was significantly higher (Figure 1c, histogram 5). This indicates that the mitochondria tend to accumulate in HD flies. Overexpression of PINK1 in HD flies restored mitochondrial area, number and density to control values (Figure 1c, histograms 3–5).

### PINK1 overexpression rescues HD neuronal pathology

**Figure 2:**
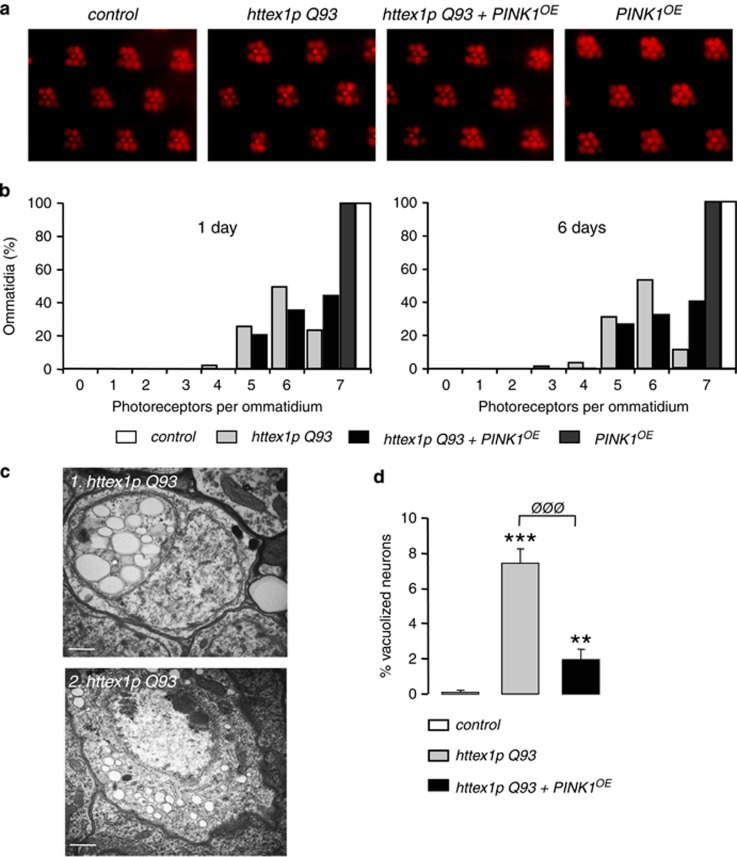
*Drosophila* PINK1 overexpression rescues neurodegeneration in flies expressing *httex1p Q93* in neurons. (**a**) Pseudopupil images from 1-day-old flies expressing no transgene (control), *httex1p Q93* alone, *httex1p Q93* together with *Drosophila PINK1* (*PINK1*^*OE*^) or *PINK1*^*OE*^ alone under the pan-neuronal driver *elav-GAL4*. In control flies, seven photoreceptors are visible, whereas the expression of *httex1p Q93* induces photoreceptor neuron loss. *PINK1*^*OE*^ counteracts the *httex1p Q93*-induced photoreceptor degeneration. (**b**) Number of photoreceptors per ommatidium in flies expressing no transgene (control), *httex1p Q93*, *httex1p Q93+PINK1*^*OE*^ or *PINK1*^*OE*^ alone at day 1 or 6 after eclosion. Significant rescue of neurodegeneration was observed at both ages when PINK1 was overexpressed in HD flies (*n*≥10 flies; *P*<0.0001, Mann–Whitney *U*-test). (**c**) Transmission electron microscopy images showing highly vacuolized neurons in 12-day-old flies expressing *httex1p Q93* in neurons. Scale bar: 50 nm. (**d**) Percentage of vacuolized degenerative neurons in 12-day-old flies expressing no transgene (control), *httex1p Q93* alone or together with *PINK1*^*OE*^. *PINK1*^*OE*^ significantly reduces mHtt-induced neurodegeneration (Student's *t*-test, **P<0.01, ***P<0.001 *versus* control; ^ØØØ^*P*<0.001 *versus* *httex1p Q93*). Quantifications were performed as followed: *n*>150 neurons in six flies per condition

Next, we determined whether PINK1 mitigates photoreceptor neurodegeneration in the retina. Pseudopupil analysis revealed that PINK1 overexpression (*PINK1*^*OE*^) markedly reduced photoreceptor loss in 1-day-old flies (Figures 2a and b). Whereas only 23% of ommatidia remained intact with seven visible photoreceptors in HD flies, this percentage reached 44% in flies coexpressing mHtt and PINK1. Similar neuroprotection was found in 6-day-old flies (Figure 2b). PINK1 overexpression in control flies had no impact on ommatidia even at 1 month of age. We also explored the effect of PINK1 on postmitotic neurons in the central brain. Although HD flies at 12 days displayed about 7% of degenerative neurons showing cytoplasmic vacuolization in the olfactive lobes (Figure 2c), <2% of vacuolized neurons were detected in the presence of PINK1 (Figure 2d).

**Figure 3:**
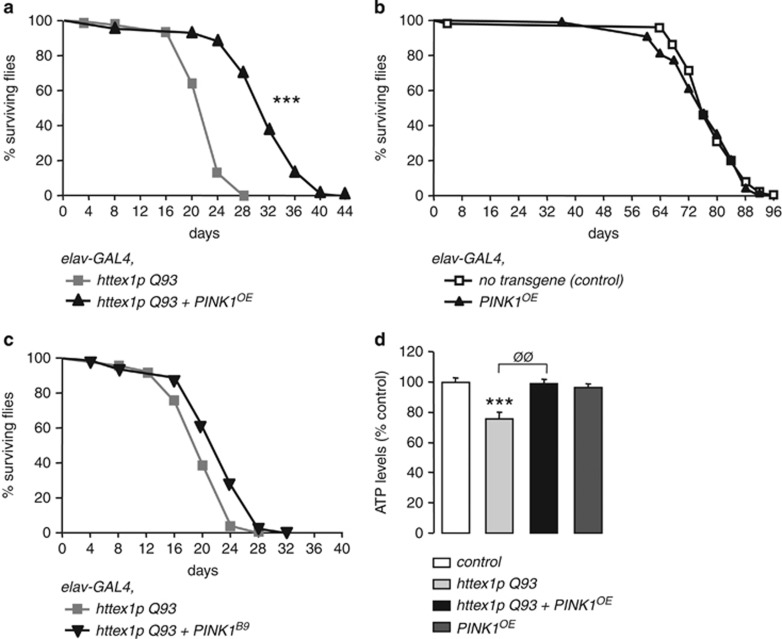
*Drosophila* PINK1 overexpression ameliorates survival of flies expressing *httex1p Q93* in neurons. (**a**) Survival rates of flies expressing *httex1p Q93* alone or together with *PINK1*^*OE*^ in neurons. Statistical significance was determined by the log-rank test (*n*>80 flies per condition; ***P<0.0001). (**b**) Survival rates of flies expressing no transgene or *PINK1*^*OE*^ under the control of the neuronal driver *elav-GAL4*. No significant change in survival is detected (*n*>60 flies per genotype). (**c**) Survival rates of flies expressing *httex1p Q93* alone or in the presence of one mutant allele of *PINK1* (*PINK1*^*B9*^) in neurons. Lifespan of HD flies expressing *PINK1*^*B9*^ was not significantly different from those expressing *httex1p Q93* alone (*n*>80 flies per genotype). (**d**) ATP levels of 7-day-old flies expressing no transgene (control), *httex1p Q93*, *httex1p Q93+PINK1*^*OE*^ or *PINK1*^*OE*^ alone. Data from at least five independent experiments were averaged and were presented as mean±S.E.M. Statistical analysis was performed using a Student's *t*-test (***P<0.001 *versus* control; ^ØØ^*P*<0.01 *versus* *httex1p Q93*)

It is well documented that mHtt neuronal expression triggers the premature death of adult flies. We found that PINK1 significantly increases both mean and median survival (+40%) of HD flies (Figure 3a). By contrast, PINK1 had no impact on the longevity of control flies (Figure 3b). On the other hand, reducing PINK1 endogenous expression in HD flies by the mean of an heterozygous PINK1 loss-of-function allele (*PINK1*^*B9*^) did not decrease HD fly lifespan (Figure 3c). The impact of *PINK1*^*B9*^ homozygosity was not tested, as *Drosophila* carrying two *PINK1*^*B9*^ mutant alleles already show decreased lifespan.^13^ We next monitored ATP levels to evaluate mitochondrial function. We found that 7-day-old flies expressing mHtt in neurons exhibited a 24% reduction of ATP levels in the brain (Figure 3d). In contrast, HD flies overexpressing PINK1 did not show decreased ATP levels at the same age (Figure 3d).

### PINK1 neuroprotection against mHtt toxicity is dependent on Parkin

**Figure 4:**
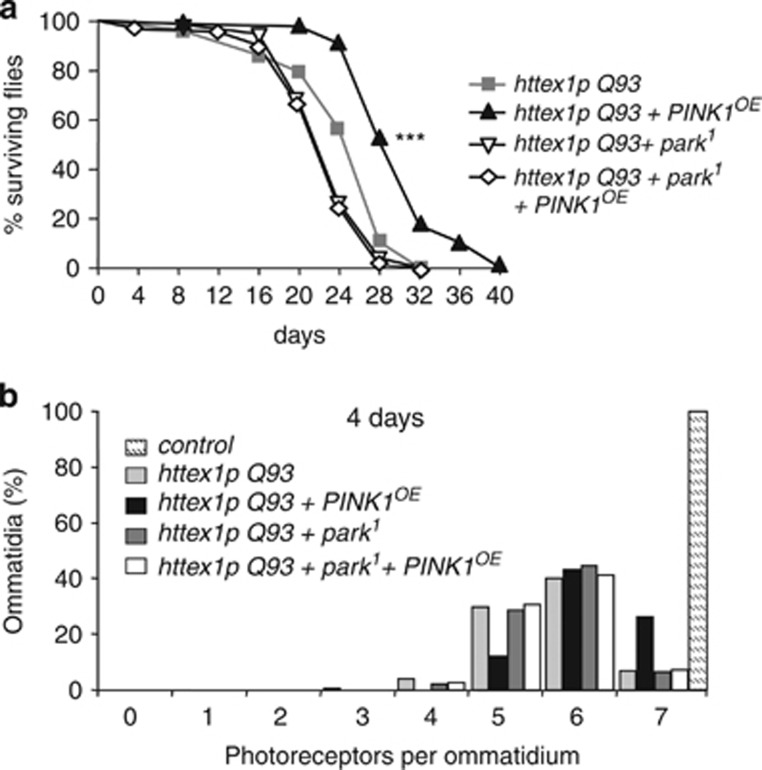
Loss of function of Parkin suppresses *Drosophila* PINK1 neuroprotective effect in HD flies. (**a**) Survival rates of flies expressing *httex1p Q93* alone, *httex1p Q93* with *PINK1*^*OE*^, *httex1p Q93* in the presence of one mutant allele of *parkin* (*park*^*1*^) or *httex1p Q93* together with *PINK1*^*OE*^ and *park*^*1*^. Loss of function of Parkin reduces the PINK1 neuroprotective effect on the survival of HD flies. Statistical significance was determined by the log-rank test (*n*>80 flies per genotype; ***P<0.0001). (**b**) Number of photoreceptors per ommatidium in 4-day-old flies expressing no transgene (control), *httex1p Q93* alone, *httex1p Q93* with *PINK1*^*OE*^, *httex1p Q93* with *park*^*1*^ or *httex1p Q93* together with *PINK1*^*OE*^ and *park*^*1*^ in neurons. Loss of function of Parkin abolishes the PINK1-mediated rescue of photoreceptor loss in HD flies (*n*=10–12 flies per genotype; *P*<0.0001, Mann–Whitney *U*-test)

To determine if PINK1-mediated neuroprotective effect involves Parkin, we examined whether *Drosophila parkin* overexpression (*parkin*^*OE*^) alleviates HD phenotype in *Drosophila*. Unexpectedly, overexpressing Parkin failed to modify the life expectancy or the photoreceptor loss of flies expressing Httex1p Q93 under the control of the neuronal driver *elav-GAL4* (Supplementary Figure S1). Coexpression of Parkin with PINK1 was deleterious as no adult flies emerged from pupae. This observation is in agreement with previous data showing that coexpression of PINK1 and Parkin under the heat-shock-GAL4 driver leads to pupal lethality.^14^ Next, we determined if partial loss of function of Parkin can prevent rescue by PINK1. We generated HD flies overexpressing PINK1 and carrying a heterozygous Parkin loss-of-function allele (*park*^*1*^). In those flies, *PINK1*^*OE*^ failed to increase lifespan (Figure 4a). We also demonstrated that PINK1 neuroprotection of photoreceptors was dependent on Parkin (Figure 4b). At 4 days, flies expressing mHtt alone exhibited 86% of ommatidia with 5–6 photoreceptors and only 9% of intact ommatidia (Figure 4b). As shown previously, PINK1 markedly reduced photoreceptor degeneration as 32% of the ommatidia remained intact. However, when *PINK1*^*OE*^ was combined with the *park*^*1*^ mutation, the neuroprotection was abolished as 88% of ommatidia contained 5–6 photoreceptors and only 9% of ommatidia were intact. No significant change on both survival and photoreceptor number was observed in control *park*^*1*^ heterozygous flies. Therefore, we demonstrate that overexpressing PINK1 provides neuroprotection against mHtt through the Parkin pathway. However, our data also show that Parkin by itself is inefficient against mHtt toxicity.

### PINK1 neuroprotective effect requires mitofusin

**Figure 5:**
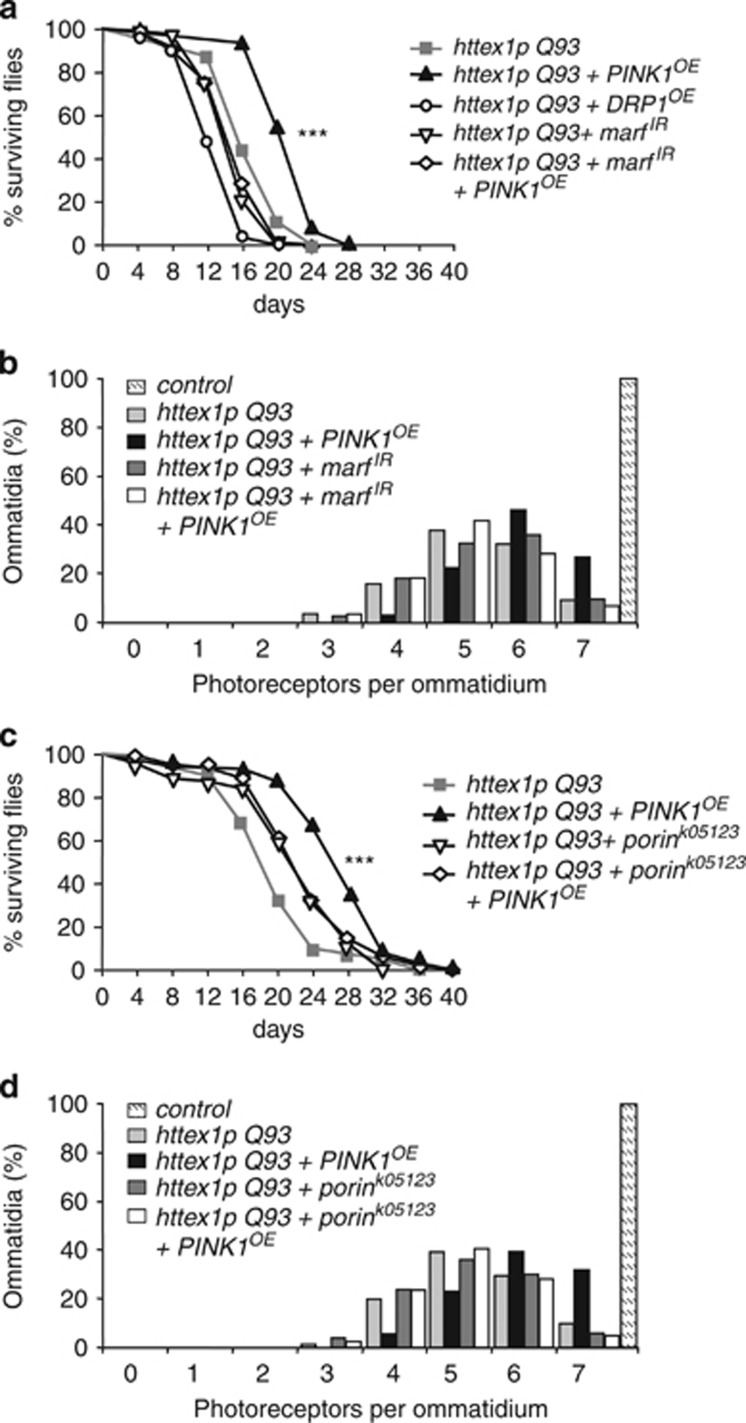
Decreasing Mfn/Marf and VDAC/Porin reduce PINK1 neuroprotective effect. (**a**) Survival rates of flies expressing *httex1p Q93* alone, *httex1p Q93* with RNA interference (RNAi) against *mfn/marf* (*marf*^*IR*^), *httex1p Q93* with a DRP1 transgene (*DRP1*^*OE*^), *httex1p Q93* with *PINK1*^*OE*^ or *httex1p Q93* together with *PINK1*^*OE*^ and *marf*^*IR*^ in neurons. Silencing Mfn/Marf reduces the PINK1 neuroprotective effect on the survival of HD flies. Statistical significance was determined by the log-rank test (*n*>60 flies per genotype; ***P<0.0001). (**b**) Number of photoreceptors per ommatidium in 4-day-old flies expressing no transgene (control), *httex1p Q93* alone, *httex1p Q93* with *PINK1*^*OE*^, *httex1p Q93* with the RNAi transgene *marf*^*IR*^ or *httex1p Q93* together with *PINK1*^*OE*^ and *marf*^*IR*^ in neurons (*n*=7–10 flies per genotype; *P*<0.0001, Mann–Whitney *U*-test). (**c**) Survival rates of flies expressing *httex1p Q93* alone, *httex1p Q93* with *PINK1*^*OE*^, *httex1p Q93* in the presence of one mutant allele of *VDAC/porin* (*porin*^*k05123*^) or *httex1p Q93* together with *PINK1*^*OE*^ and *porin*^*k05123*^. Loss of function of Porin prevents the PINK1 neuroprotective effect on the survival of HD flies. Statistical significance was determined by the log-rank test (*n*>60 flies per genotype; ***P<0.0001). (**d**) Number of photoreceptors per ommatidium in 4-day-old flies expressing no transgene (control), *httex1p Q93* alone, *httex1p Q93* with *PINK1*^*OE*^, *httex1p Q93* with the mutant allele *porin*^*k05123*^ or *httex1p Q93* together with *PINK1*^*OE*^ and *porin*^*k05123*^ in neurons. Loss of function of Porin reduces the PINK1-mediated rescue of photoreceptor loss in HD flies (*n*=6 flies per genotype; *P*<0.0001, Mann–Whitney *U*-test)

The mechanisms by which PINK1/Parkin pathway mediates phagophore formation and mitophagy are still elusive. Mitofusin (Mfn) is a target of Parkin and its degradation might promote mitochondrial fission and facilitate mitochondrial removal.^15^ We then analyzed whether or not increasing mitochondrial fission could influence mHtt toxicity. We found that RNAi silencing of the *Drosophila mfn* homolog, *marf* (*marf*^*IR*^), or overexpressing *Drosophila* fission effector, *DRP1* (*DRP1*^*OE*^), failed to increase the lifespan of flies expressing mHtt in neurons (Figure 5a). The survival rate of HD flies was even reduced by about 23% in the presence of *DRP1*^*OE*^. We also determined whether Mfn/Marf inactivation could impact the neuroprotective effect of PINK1. Whereas overexpressing PINK1 increased the life expectancy of HD flies, no significant effect was detected when *marf*^*IR*^ was coexpressed with PINK1 (Figure 5a). We then analyzed the impact of Mfn/Marf inactivation on the loss of photoreceptors (Figure 5b). Interestingly, while silencing Mfn/Marf did not affect neuronal loss in HD fly retina, we observed a significant reduction of the PINK1-mediated neuronal rescue. Our data suggest that Mfn/Marf is involved in PINK1-mediated neuroprotection.

### VDAC/Porin is necessary for PINK1-mediated rescue

It has been proposed that voltage-dependent anion channel (VDAC) could be part of the PINK1/Parkin pathway.^16, 17^ As shown in Figure 5c, the presence of one mutant allele of *porin* (*porin*^*k05123*^), the *Drosophila* homologous of VDAC, was sufficient to counteract PINK1-mediated rescue on the survival of HD flies. Whereas flies coexpressing mHtt and PINK1 in neurons showed a 35% increase in life expectancy *versus* flies expressing mHtt alone, PINK1 was uneffective on HD flies carrying one copy of *porin*^*k05123*^. The loss of function of VDAC/Porin led by itself to a very slight amelioration of the survival of HD flies, which could be related to the direct role of VDAC in the apoptotic release of mitochondrial cytochrome *C*. We also tested the impact of VDAC/Porin on the loss of photoreceptor neurons induced by mHtt (Figure 5d). Whereas the presence of one copy of *porin*^*k05123*^ allele did not affect neuronal loss, *porin*^*k05123*^ significantly reduced the neuronal rescue by PINK1. Thus, we propose that PINK1 neuroprotection against mHtt toxicity also involves VDAC/Porin.

### TRAP1 is not required for PINK1 neuroprotective effect

In parallel of PINK1/Parkin-mediated mitophagy, PINK1 can also regulate mitochondrial integrity by direct activation of mitochondrial chaperones such as TNF receptor-associated protein 1 (TRAP1).^18, 19^ We found that increasing TRAP1 expression failed to reduce the organismal death of HD flies (Supplementary Figure S2). Therefore, PINK1 neuroprotection is likely not dependent on the activation of TRAP1 in the HD context.

### The presence of mHtt compromises the degradation of defective mitochondria in mouse striatal cells

**Figure 6:**
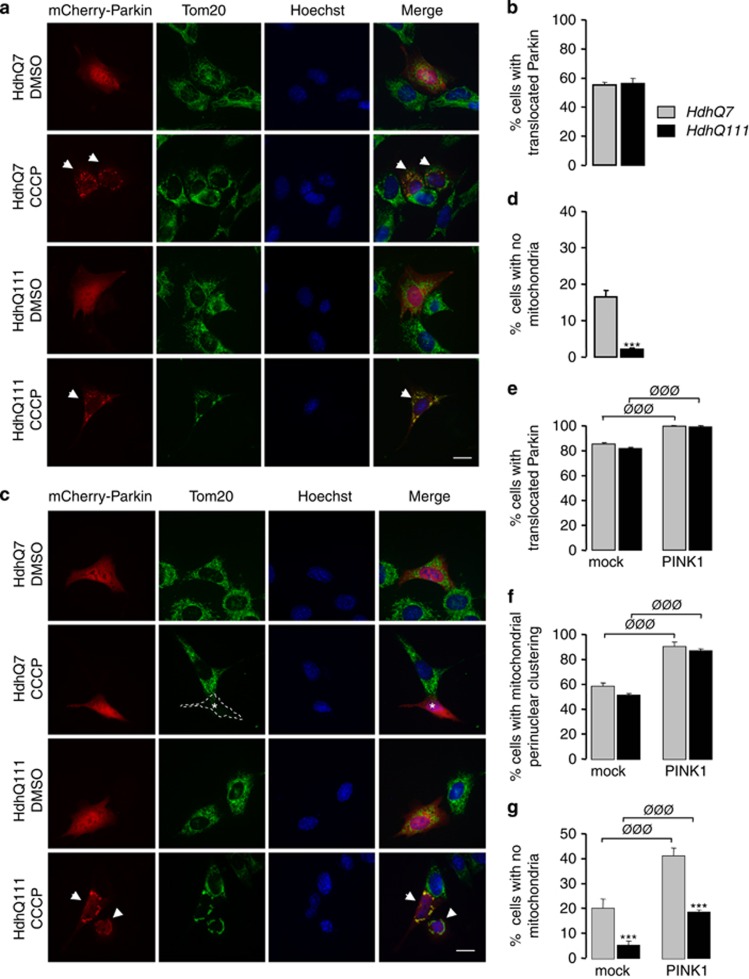
Parkin-directed mitophagy is altered by mHtt in HdhQ111 striatal cells but is partially restored by PINK1. (**a**) Striatal cells derived from HdhQ7 and HdhQ111 knock-in mice were transfected with mCherry-Parkin and treated by dimethyl sulfoxide (DMSO) or 10 *μ*M CCCP for 6 h. Mitochondria were immunostained by an anti-Tom20 antibody and nuclei were visualized by Hoechst stain. Arrowheads show translocation of mCherry-Parkin to the mitochondria in CCCP-treated cells. Scale bar: 10 *μ*m. (**b**) Quantitative analyses of the percentage of cells with Parkin translocated to the mitochondria. Data from at least three independent experiments (3–6 coverslips each) were averaged and were presented as mean±S.E.M. (**c**) HdhQ7 and HdhQ111 striatal cells were transfected with mCherry-Parkin and treated by DMSO or 10 *μ*M CCCP for 38 h. Mitochondria were immunostained by an anti-Tom20 antibody and nuclei were visualized by Hoechst stain. Arrowheads show cells with Parkin translocation and perinuclear mitochondrial clustering. White asterisk corresponds to a CCCP-treated cell devoid of Tom20-immunostained mitochondria. Scale bar: 10 *μ*m. (**d**) Quantitative analyses of the percentage of cells with no Tom20-immunostained mitochondria. Data from at least three independent experiments (3–6 coverslips each) were averaged and were presented as mean±S.E.M. Statistical analysis was performed using a Student's *t*-test (***P<0.001 *versus* HdhQ7 cells). (**e**–**g**) Quantitative analyses of the percentage of cells showing Parkin translocation to the mitochondria (**e**), the proportion of cells showing perinuclear clustering of the mitochondria (**f**) or cells with no mitochondria (**g**). HdhQ7 and HdhQ111 striatal cells were transfected with mCherry-Parkin together with mock plasmid or PINK1-HA and treated with 10 *μ*M CCCP for 38 h. Mitochondria were detected by Tom20 immunostaining. Data from at least three independent experiments (3–6 coverslips each) were averaged and were presented as mean±S.E.M. Statistical analysis was performed using a Student's *t*-test (^ØØØ^*P*<0.001 PINK1-transfected *versus* mock-transfected cells; ***P<0.001 *versus* HdhQ7 cells)

One important question raised by our data is whether or not mHtt may directly impact mitophagy. To investigate this issue, we used mouse striatal HdhQ7 and HdhQ111 cell lines that were immortalized from knock-in mice carrying 7 and 111 CAG, respectively, in the mouse *htt* gene.^20^ As those cells did not strongly express Parkin (data not shown), they were transfected with Parkin plasmids. Parkin is specifically recruited to the mitochondria in response to dissipation of mitochondrial membrane potential. Accordingly, treatment with the mitochondrial uncoupler, carbonyl cyanide *m*-chlorophenyl hydrazone (CCCP; 10 *μ*M for 6 h) led to the translocation of transfected mCherry-Parkin from the cytoplasm to Tom20-immunostained mitochondria, whereas striatal cells treated with DMSO exhibited diffuse localization of mCherry-Parkin in the cytoplasm (Figure 6a). We found that 55% of HdhQ7 striatal cells showed Parkin translocation after CCCP treatment (Figure 6b). Importantly, mHtt in HdhQ111 did not modify the proportion of cells showing Parkin translocation to the mitochondria. As previously observed in HeLa cells,^16, 21^ striatal cells treated with CCCP for a longer period (10 *μ*M for 38 h) exhibited a clustering of their mitochondria around the nucleus, followed by subsequent clearance of mitochondria as revealed by the loss of Tom20 staining (Figure 6c). However, strikingly, whereas 17% of HdhQ7 cells were devoid of Tom20-immunostained mitochondria, this amount fell down to only 2% for HdhQ111 cells (Figure 6d). At the same time, no change was detected between HdhQ7 and HdhQ111 cells regarding the capacity of Parkin to translocate to the mitochondria (Figure 6e) or perinuclear mitochondrial clustering (Figure 6f). We also confirmed these observations by using another mitochondrial marker, Tim23, which stains the inner membrane (Supplementary Figure S3). Altogether, our data indicate that mHtt could perturb the mitophagy process, likely downstream of Parkin translocation and mitochondrial perinuclear clustering.

### PINK1 overexpression is able to ameliorate mitophagy process in mouse striatal cells

As mHtt compromises mitophagy, we determined whether PINK1 overexpression was still able to boost mitophagy in HdhQ111 cells. After 38 h CCCP treatment, PINK1 transfection increased the proportion of HdhQ7 and HdhQ111 cells showing Parkin mitochondrial translocation (Figure 6e) or mitochondrial perinuclear clustering (Figure 6f). More importantly, PINK1 overexpression increased the number of HdhQ7 and HdhQ111 cells with no detectable mitochondria (Figure 6g). However, the number of cells with no Tom20-immunostained mitochondria remained reduced for HdhQ111 *versus* HdhQ7 cells, further confirming that mHtt impacts mitophagy. Similar results were obtained when Tim23 immunostaining was used to visualize mitochondria (Supplementary Figure S3). Thus, our data show that overexpressing PINK1 is still able to increase partially the removal of defective mitochondria even in the presence of mHtt.

### The targeting of mitochondria to autophagosomes is decreased by mHtt

**Figure 7:**
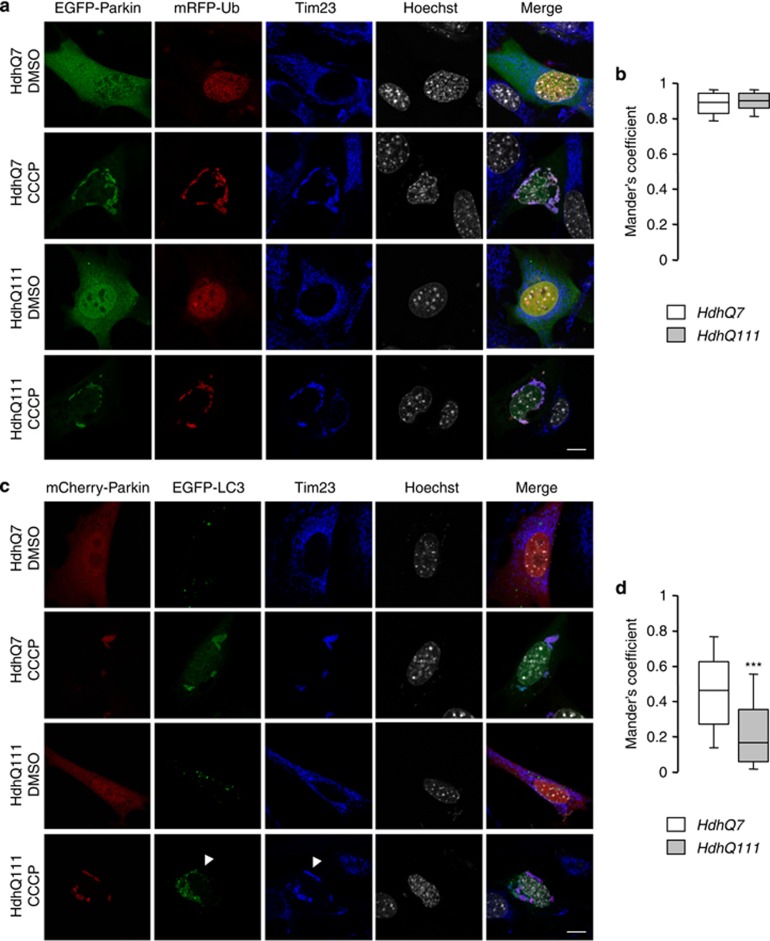
The presence of mHtt alters the formation of mitochondria-containing autophagosomes. (**a**) HdhQ7 and HdhQ111 striatal cells were transfected with EGFP-Parkin, PINK1-HA and mRFP-ubiquitin (mRFP-Ub), and then treated by dimethyl sulfoxide (DMSO) or 10 *μ*M CCCP for 38 h. Mitochondria were visualized by anti-Tim23 immunostaining. Scale bar: 10 *μ*m. (**b**) Quantitative analyses showing the Mander's colocalization coefficient between mRFP-ubiquitin and mitochondria. Data from at least 30 cells from six coverslips were averaged and were presented as mean±S.E.M. (**c**) Striatal cells were transfected with mCherry-Parkin, PINK1-HA and EGFP-LC3, and then treated by DMSO or 10 *μ*M CCCP for 38 h. Mitochondria were visualized by anti-Tim23 immunostaining. Arrowheads show mitochondria that are not colocalized with EGFP-LC3 in CCCP-treated cells. Scale bar: 10 *μ*m. (**d**) Quantitative analyses showing the Mander's colocalization coefficient between EGFP-LC3 and mitochondria. Data from at least 30 cells from six coverslips were averaged and were presented as mean±S.E.M. Statistical analysis was performed using the Mann–Whitney *U*-test (***P<0.001 *versus* HdhQ7 cells)

Next, we further investigated at which step mitophagy process may be compromised by mHtt. Parkin promotes mitophagy by triggering mitochondrial ubiquitination, which in turn recruits ubiquitin-binding autophagic components. As shown in Figure 7a, CCCP treatment (10 *μ*M for 38 h) strongly increased the ubiquitination of Tim23-immunostained mitochondria as compared with DMSO. Quantitative analysis revealed no change in the ubiquitination rate of CCCP-treated mitochondria between HdhQ111 and HdhQ7 cells (Figure 7b; Mander's coefficients: 0.88±0.01 and 0.89±0.01 for HdhQ7 and HdhQ111 cells, respectively). Treatment with CCCP also induced the accumulation of LC3-positive autophagic vesicles in striatal cells (Figure 7c). Interestingly, colocalization between GFP-tagged LC3 and mitochondria was significantly reduced in the HdhQ111 *versus* HdhQ7 cells (Figure 7d; Mander's coefficients: 0.45±0.02 and 0.23±0.01 for HdhQ7 and HdhQ111 cells, respectively). This suggests that mHtt impairs mitophagy, likely by perturbing the targeting of defective mitochondria to autophagosomes.

## Discussion

Our data demonstrate that enhancing mitochondrial quality control by overexpressing PINK1 confers protection against mHtt toxicity. Indeed, PINK1 overexpression rescues abnormal mitochondrial spheroid formation, neuronal loss, ATP levels and, more importantly, it increases the survival of adult flies expressing mHtt in neurons. Additionally, we have dissected the mechanisms underlying PINK1-mediated neuroprotection. We showed that it requires the E3 ubiquitin ligase Parkin and the outer membrane mitochondrial proteins Mfn/Marf and VDAC/Porin. Furthermore, we provided the first evidence that HD mutation affects mitophagy in HD striatal cells derived from HdhQ111 knock-in mice. Thus, our findings highlight the potential role played by damaged mitochondria accumulation in HD pathogenesis, and support the PINK1/Parkin pathway as a valuable therapeutic target.

Many studies have described abnormal mitochondrial fragmentation and cristae disruption in HD models and patients.^4, 5, 6, 22, 23^ It was proposed that increased fission might have a direct role in HD pathogenesis. In agreement with this, reducing DRP1 activity protected cultured neurons expressing mHtt from axonal trafficking defects and cell death,^6^ and rescued motility defects in a *Caenorhabditis elegans* model of HD.^22^ More recently, a selective inhibitor of DRP1, P110-TAT, was shown to inhibit the pathology of HD mouse models.^24^ It was also reported that mHtt directly interacts with DRP1 and, thereby, increases DRP1 GTPase activity and its association with mitochondria.^6, 23, 24^ This may explain why we found that increasing DRP1 expression in our HD fly model exacerbates further the adult lethality.

In the present study, we detected the presence of abnormal mitochondria that adopt a ring-like form, termed mitochondrial spheroids. Such mitochondrial shape was rarely observed *in vivo* and their significance in pathological conditions remains to be fully investigated. So far, they were reported in injured livers of acetaminophen-overdosed mice and in retinal lesions of chicks reared in continuous light, suggesting that mitochondrial spheroids may appear in specific cell types upon cellular stress.^10, 25^ More importantly, mitochondrial spheroids were found *in vitro* after treating Parkin-deficient murine embryonic fibroblasts and HeLa cells with the mitochondrial uncoupler CCCP, an ionophore that usually induces mitophagy.^10^ In this study, Ding *et al.*^10^ showed that mitochondria engage in a self-fusion process when mitophagy is defective. Moreover, reactive oxygen species seem to be required since antioxidants suppress mitochondrial spheroid formation even in the presence of CCCP.^10^ Taking those data into account, our study suggests that the mitophagy pathway might be overwhelmed by the accumulation of damaged mitochondria in the presence of mHtt, leading to the formation of mitochondrial spheroids. This is consistent with mitochondrial spheroid formation being reduced when we overexpressed PINK1, which controls the activation of Parkin-mediated mitophagy. Nevertheless, it is also possible that mHtt directly inhibits the mitophagy process, leading to the accumulation of defective mitochondria. In support of this hypothesis, we found that the presence of mHtt in mammalian HdhQ111 striatal cells perturbs CCCP-induced mitophagy but not Parkin translocation. Thus, HD mutation likely alters mitophagy downstream of Parkin translocation. Previous studies reported that mHtt could sequester proautophagic proteins and that there is a failure in the recognition of cargo by autophagosomes.^26, 27^ Our data also provide evidence that formation of mitochondria-containing autophagosomes is disrupted by mHtt in HdhQ111 striatal cells. More recently, autophagosome transport was also found defective in primary neurons expressing mHtt, leading to inefficient degradation of engulfed mitochondria.^28^ We show here that whereas HD mutation compromised mitophagy, PINK1 overexpression was still able to increase partially the removal of defective mitochondria in HdhQ111 cells. It is thus tempting to propose that overexpressing PINK1 might decrease the threshold of mitochondrial damage that must be crossed before mitophagy occurs. Then, PINK1 might accelerate the removal of defective mitochondria against the progressive impairment of autophagy.

Previous studies have reported that PINK1 overexpression is neuroprotective in the context of PD. PINK1 prevents cell death induced by the parkinsonian neurotoxin 1-methyl-4-phenyl-1,2,3,6-tetrahydropyridine^29^ or apoptotic inducers in cell cultures.^30^ Similarly, overexpression of PINK1 ameliorates the lifespan and mobility of *Drosophila* expressing the PD-related gene, *α*-synuclein, in dopamine neurons.^31^ Mitochondrial stress mechanisms are not restricted to PD but are common to other neurodegenerative diseases. We provide here, for the first time, compelling evidence that PINK1 pathway mitigates neuronal loss and increases life expectancy in an animal model of HD.

PINK1 led to intensive *in vitro* studies to elucidate how it controls mitochondrial quality control. The mitochondrial chaperone TRAP1 might be a direct substrate of PINK1, allowing the refolding/removal of misfolded proteins and preventing apoptotic cytochrome *C* release.^18^ However, our data suggest that TRAP1 overexpression does not mimic PINK1-mediated protection in HD flies. At the organelle level, PINK1 facilitates mitophagy through the recruitment of Parkin to dysfunctional mitochondria.^7, 8, 9^ Moreover, direct phosphorylation of Parkin at Ser65 by PINK1 seems to be required to initiate mitophagy.^32, 33, 34^ Then, Parkin might tag outer membrane mitochondrial substrates for proteasomal degradation and/or for autophagosome recognition. We here demonstrated that whereas Parkin is essential to mediate PINK1 neuroprotection, overexpressing Parkin alone was ineffective to confer protection against mHtt toxicity. This is in agreement with the crucial role of PINK1 in determining which mitochondria must be removed by mitophagy. Moreover, ubiquitination of Mfn by Parkin may account for its rapid removal from the mitochondria, therefore preventing damaged mitochondrial fragments to fuse back and corrupt healthy mitochondria.^35, 36^ However, in our study, silencing Mfn failed to ameliorate the survival of HD flies and abolished the beneficial effect of PINK1 on neuronal survival. Mfn degradation is therefore not a sufficient condition to mimic PINK1 effect. On the contrary, our data suggest that normal Mfn expression is required for PINK1-mediated neuroprotection. This is consistent with recent data which demonstrate that in mammals Mfn2 is phosphorylated by PINK1 and promotes PINK1-dependent translocation of Parkin to the mitochondria.^37^ Finally, we also showed that VDAC/Porin is involved in PINK1-mediated reduction of mHtt toxicity in *Drosophila*. Whether or not VDAC is necessary for PINK1/Parkin pathway remains controversial. Mammalian VDAC1 was identified as a target for Parkin-mediated ubiquitination and to promote mitophagy in HeLa cells.^16^ In contrast, while ubiquitination of VDAC1 was confirmed in MEF cells with damaged mitochondria, VDAC1 appeared dispensable for mitochondrial clustering and mitophagy in those cells.^21^ The role of VDAC in mitophagy may be redundant and depend on cell types. More recently, it was hypothesized that VDAC may serve as a docking site to recruit Parkin from the cytosol to defective mitochondria.^17^ Finally, PINK1 could interact with the proautophagic protein Beclin1, which is also sequestered by mHtt.^26^ Further studies are required to fully understand in which conditions and how Mfn and VDAC participate in PINK1/Parkin pathway.

In conclusion, we propose that mitophagy may reach a saturation stage in the presence of mHtt and that increasing PINK1/Parkin pathway may improve mitochondrial integrity in HD. It is therefore conceivable that PINK1/Parkin pathway may be of therapeutic interest not only in PD but also in other mitochondrial disorders.

## Materials and Methods

### *Drosophila* strains

Flies were raised on a standard agar/cornmeal/yeast diet at 25 °C. Fly stocks carrying *PINK1*^*B9*^, *PINK1*^*OE*^ *and parkin*^*OE*^ were from Pr. J Chung (Taejon, Korea), flies carrying *DRP1*^*OE*^ and *marf*^*IR*^ from Dr. M Guo (Los Angeles, CA, USA), flies carrying *TRAP1*^*OE*^ (lines 1 and 2 M) from Dr. LM Martins (Leicester, UK). All other strains were obtained from the Bloomington *Drosophila* Stock Center (Bloomington, IN, USA). In accordance with the genetic background, we used *w*^*1118*^ flies as controls.

### Electron microscopy

Adult fly heads were fixed in 2% paraformaldehyde, 2.5% glutaraldehyde, 5mM CaCl_2_ and 0.1mM sodium cacodylate for 24 h at 4 °C. Postfixation was performed in 2.5% glutaraldehyde, 0.8% osmium tetroxide and 0.1 mM sodium cacodylate for 2 h at 4 °C. After staining with 2% uranyl acetate and dehydratation, samples were included in Epon resin. Ultrathin sections were examined with a Philips CM10 (Eindhoven, The Netherlands) or a Leo 912 Zeiss (Zeiss Microscopy, Jena, Germany) microscope at 100 kV.

### Pseudopupil analysis

Pseudopupil analysis was performed as described previously.^12^ The number of photoreceptors was determined from 20 ommatidia per fly. Data from at least seven flies per condition were averaged and were presented as mean±S.E.M. Statistical analysis was performed using the non-parametric Mann–Whitney *U*-test.

### Longevity assays

Survival analysis was performed on female flies grouped by 15–20 in independent vials. Each genotype was evaluated at least two times. Survival curves were generated by using the GraphPad Prism software (La Jolla, CA, USA) and statistical analysis was performed by log-rank test. To establish a high-confidence list of modifier genes, we considered significant only genes that modify life expectancy for more than the lifespan variance of Httex1p Q93-expressing flies between tubes within each trial.

### Measurement of ATP levels

ATP measurements were essentially performed according to the manufacturer's instructions (Luminescent ATP Detection Assay Kit; Abcam, Cambridge, UK). For each experiment, five adult fly brains were rapidly dissected, and the extracts were immediately deproteinized by perchloric acid precipitation. Luminescence was measured with a TriStar LB941 luminometer (Berthold Technologies, Bad Wildbad, Germany). Data from at least five independent experiments were averaged and were presented as mean±S.E.M. Statistical analysis was performed using a Student's *t*-test.

### Striatal cell culture, transfection and CCCP treatment

Analysis of mitophagy was performed on striatal cell lines derived from wild-type and mHtt knock-in mice: HdhQ7 and HdhQ111 (from Coriell Cell Repositories, Camden, NJ, USA).^20^ Cells were grown in Dulbecco's modified Eagle's medium (Invitrogen, Carlsbad, CA, USA) supplemented with 10% fetal bovine serum (Invitrogen), penicillin, streptomycin and G418 (Invitrogen) and maintained at 33 °C in a 5% CO_2_ incubator. Striatal cells were seeded at 100 000 cells per coverslip density in 12-well plates for immunostaining. Transfection with mCherry-Parkin (generous gift from Richard J Youle, Bethesda, MD, USA), EGFP-Parkin (generous gift from Wolfdieter Springer, Tübingen, Germany), PINK1-HA (generous gift from Ricardo Vago, Milan, Italy), EGFP-LC3 (Addgene, Cambridge, MA, USA) or mRFP-Ubiquitin (Addgene) plasmids was performed using Lipofectamine (Invitrogen) according to the manufacturer's instructions. One day after transfection, dissipation of mitochondrial membrane potential was achieved with 10 *μ*M CCCP (prepared in DMSO) for 6 or 38 h.

### Immunostaining

HdhQ7 and HdhQ111 striatal cells were washed with phosphate-buffered saline (PBS; Invitrogen), fixed in 4% paraformaldehyde for 10 min, permeabilized in 0.1% Triton X-100 in PBS for 10 min and incubated for 2 h at room temperature with anti-Tom20 (Santa Cruz Biotechnology, Dallas, TX, USA) or anti-Tim23 antibodies (BD Biosciences, Franklin Lakes, NJ, USA) or anti-HA antibodies for PINK1 detection (Roche, Meylan, France) diluted in 3% bovine serum albumin. Then, cells were incubated for 45 min with secondary antibodies prepared in 3% bovine serum albumin and mounted in Mowiol (Calbiochem, La Jolla, CA, USA). Quantitative analyses were achieved using an AX10 Zeiss Apotome (Zeiss Microscopy). Proportion of cells showing mitochondrial Parkin translocation, mitochondrial perinuclear clustering or with no detectable mitochondrial markers were evaluated on at least 70 cells per coverslip. Data from three independent experiments with 3–6 coverslips each were averaged and were presented as mean±S.E.M. Statistical analysis was performed using a Student's *t*-test. Confocal image acquisition was performed on a Zeiss LSM780 laser scanning (Zeiss Microscopy) microscope. Colocalization between mRFP-ubiquitin or EGFP-LC3 with Tim23-immunostained mitochondria was evaluated on a minimum of 30 cells from six coverslips. Mander's colocalization coefficient was calculated with the JACoP plugin of the Image J software. Data were presented as mean±S.E.M. and statistical analysis was performed using the non-parametric Mann–Whitney *U*-test.

## Abbreviations

CCCP: carbonyl cyanide *m*-chlorophenyl hydrazone
DRP1: dynamin-related protein 1
HD: Huntington's disease
Mfn: mitofusin
mHtt: mutant Huntingtin
PD: Parkinson's disease
PGC-1*α*: peroxisome proliferator-activated receptor gamma coactivator-1-alpha
PINK1: PTEN-induced putative kinase 1
TRAP1: TNF receptor-associated protein 1
VDAC: voltage-dependent anion channel

## References

[bib1] Browne SE, Bowling AC, MacGarvey U, Baik MJ, Berger SC, Muqit MM et al. Oxidative damage and metabolic dysfunction in Huntington's disease: selective vulnerability of the basal ganglia. Ann Neurol 1997; 41: 646–653. 9153527 10.1002/ana.410410514

[bib2] Gu M, Gash MT, Mann VM, Javoy-Agid F, Cooper JM, Schapira AH. Mitochondrial defect in Huntington's disease caudate nucleus. Ann Neurol 1996; 39: 385–389. 8602759 10.1002/ana.410390317

[bib3] Cui L, Jeong H, Borovecki F, Parkhurst CN, Tanese N, Krainc D. Transcriptional repression of PGC-1alpha by mutant huntingtin leads to mitochondrial dysfunction and neurodegeneration. Cell 2006; 127: 59–69. 17018277 10.1016/j.cell.2006.09.015

[bib4] Kim J, Moody JP, Edgerly CK, Bordiuk OL, Cormier K, Smith K et al. Mitochondrial loss, dysfunction and altered dynamics in Huntington's disease. Hum Mol Genet 2010; 19: 3919–3935. 20660112 10.1093/hmg/ddq306 PMC2947400

[bib5] Shirendeb UP, Calkins MJ, Manczak M, Anekonda V, Dufour B, McBride JL et al. Mutant huntingtin's interaction with mitochondrial protein Drp1 impairs mitochondrial biogenesis and causes defective axonal transport and synaptic degeneration in Huntington's disease. Hum Mol Genet 2012; 21: 406–420. 21997870 10.1093/hmg/ddr475 PMC3276281

[bib6] Song W, Chen J, Petrilli A, Liot G, Klinglmayr E, Zhou Y et al. Mutant huntingtin binds the mitochondrial fission GTPase dynamin-related protein-1 and increases its enzymatic activity. Nat Med 2011; 17: 377–382. 21336284 10.1038/nm.2313 PMC3051025

[bib7] Matsuda N, Sato S, Shiba K, Okatsu K, Saisho K, Gautier CA et al. PINK1 stabilized by mitochondrial depolarization recruits Parkin to damaged mitochondria and activates latent Parkin for mitophagy. J Cell Biol 2010; 189: 211–221. 20404107 10.1083/jcb.200910140 PMC2856912

[bib8] Narendra DP, Jin SM, Tanaka A, Suen DF, Gautier CA, Shen J et al. PINK1 is selectively stabilized on impaired mitochondria to activate Parkin. PLoS Biol 2010; 8: e1000298. 20126261 10.1371/journal.pbio.1000298 PMC2811155

[bib9] Vives-Bauza C, Zhou C, Huang Y, Cui M, de Vries RL, Kim J et al. PINK1-dependent recruitment of Parkin to mitochondria in mitophagy. Proc Natl Acad Sci USA 2010; 107: 378–383. 19966284 10.1073/pnas.0911187107 PMC2806779

[bib10] Ding WX, Guo F, Ni HM, Bockus A, Manley S, Stolz DB et al. Parkin and mitofusins reciprocally regulate mitophagy and mitochondrial spheroid formation. J Biol Chem 2012; 287: 42379–42388. 23095748 10.1074/jbc.M112.413682 PMC3516781

[bib11] Ding WX, Li M, Biazik JM, Morgan DG, Guo F, Ni HM et al. Electron microscopic analysis of a spherical mitochondrial structure. J Biol Chem 2012; 287: 42373–42378. 23093403 10.1074/jbc.M112.413674 PMC3516780

[bib12] Liévens JC, Iche M, Laval M, Faivre-Sarrailh C, Birman S. AKT-sensitive or insensitive pathways of toxicity in glial cells and neurons in *Drosophila* models of Huntington's disease. Hum Mol Genet 2008; 17: 882–894. 18065778 10.1093/hmg/ddm360

[bib13] Park J, Lee SB, Lee S, Kim Y, Song S, Kim S et al. Mitochondrial dysfunction in *Drosophila* PINK1 mutants is complemented by parkin. Nature 2006; 441: 1157–1161. 16672980 10.1038/nature04788

[bib14] Park J, Lee G, Chung J. The PINK1-Parkin pathway is involved in the regulation of mitochondrial remodeling process. Biochem Biophys Res Commun 2009; 378: 518–523. 19056353 10.1016/j.bbrc.2008.11.086

[bib15] Narendra DP, Youle RJ. Targeting mitochondrial dysfunction: role for PINK1 and Parkin in mitochondrial quality control. Antioxid Redox Signal 2011; 14: 1929–1938. 21194381 10.1089/ars.2010.3799 PMC3078490

[bib16] Geisler S, Holmstrom KM, Skujat D, Fiesel FC, Rothfuss OC, Kahle PJ et al. PINK1/Parkin-mediated mitophagy is dependent on VDAC1 and p62/SQSTM1. Nat Cell Biol 2010; 12: 119–131. 20098416 10.1038/ncb2012

[bib17] Sun Y, Vashisht AA, Tchieu J, Wohlschlegel JA, Dreier L. Voltage-dependent anion channels (VDACs) recruit Parkin to defective mitochondria to promote mitochondrial autophagy. J Biol Chem 2012; 287: 40652–40660. 23060438 10.1074/jbc.M112.419721 PMC3504778

[bib18] Pridgeon JW, Olzmann JA, Chin LS, Li L. PINK1 protects against oxidative stress by phosphorylating mitochondrial chaperone TRAP1. PLoS Biol 2007; 5: e172. 17579517 10.1371/journal.pbio.0050172 PMC1892574

[bib19] Costa AC, Loh SH, Martins LM. *Drosophila* Trap1 protects against mitochondrial dysfunction in a PINK1/parkin model of Parkinson's disease. Cell Death Dis 2013; 4: e467. 23328674 10.1038/cddis.2012.205 PMC3563993

[bib20] Trettel F, Rigamonti D, Hilditch-Maguire P, Wheeler VC, Sharp AH, Persichetti F et al. Dominant phenotypes produced by the HD mutation in STHdh(Q111) striatal cells. Hum Mol Genet 2000; 9: 2799–2809. 11092756 10.1093/hmg/9.19.2799

[bib21] Narendra D, Kane LA, Hauser DN, Fearnley IM, Youle RJ. p62/SQSTM1 is required for Parkin-induced mitochondrial clustering but not mitophagy; VDAC1 is dispensable for both. Autophagy 2010; 6: 1090–1106. 20890124 10.4161/auto.6.8.13426 PMC3359490

[bib22] Wang H, Lim PJ, Karbowski M, Monteiro MJ. Effects of overexpression of huntingtin proteins on mitochondrial integrity. Hum Mol Genet 2009; 18: 737–752. 19039036 10.1093/hmg/ddn404 PMC2722218

[bib23] Costa V, Giacomello M, Hudec R, Lopreiato R, Ermak G, Lim D et al. Mitochondrial fission and cristae disruption increase the response of cell models of Huntington's disease to apoptotic stimuli. EMBO Mol Med 2010; 2: 490–503. 21069748 10.1002/emmm.201000102 PMC3044888

[bib24] Guo X, Disatnik MH, Monbureau M, Shamloo M, Mochly-Rosen D, Qi X. Inhibition of mitochondrial fragmentation diminishes Huntington's disease-associated neurodegeneration. J Clin Invest 2013; 123: 5371–5388. 24231356 10.1172/JCI70911 PMC3859413

[bib25] Yin XM, Ding WX. The reciprocal roles of PARK2 and mitofusins in mitophagy and mitochondrial spheroid formation. Autophagy 2013; 9: 1687–1692. 24162069 10.4161/auto.24871

[bib26] Shibata M, Lu T, Furuya T, Degterev A, Mizushima N, Yoshimori T et al. Regulation of intracellular accumulation of mutant Huntingtin by Beclin 1. J Biol Chem 2006; 281: 14474–14485. 16522639 10.1074/jbc.M600364200

[bib27] Martinez-Vicente M, Talloczy Z, Wong E, Tang G, Koga H, Kaushik S et al. Cargo recognition failure is responsible for inefficient autophagy in Huntington's disease. Nat Neurosci 2010; 13: 567–576. 20383138 10.1038/nn.2528 PMC2860687

[bib28] Wong YC, Holzbaur EL. The regulation of autophagosome dynamics by huntingtin and HAP1 is disrupted by expression of mutant huntingtin, leading to defective cargo degradation. J Neurosci 2014; 34: 1293–1305. 24453320 10.1523/JNEUROSCI.1870-13.2014 PMC3898289

[bib29] Haque ME, Thomas KJ, D'Souza C, Callaghan S, Kitada T, Slack RS et al. Cytoplasmic Pink1 activity protects neurons from dopaminergic neurotoxin MPTP. Proc Natl Acad Sci USA 2008; 105: 1716–1721. 18218782 10.1073/pnas.0705363105 PMC2234210

[bib30] Petit A, Kawarai T, Paitel E, Sanjo N, Maj M, Scheid M et al. Wild-type PINK1 prevents basal and induced neuronal apoptosis, a protective effect abrogated by Parkinson disease-related mutations. J Biol Chem 2005; 280: 34025–34032. 16079129 10.1074/jbc.M505143200

[bib31] Todd AM, Staveley BE. Pink1 suppresses alpha-synuclein-induced phenotypes in a *Drosophila* model of Parkinson's disease. Genome 2008; 51: 1040–1046. 19088817 10.1139/G08-085

[bib32] Iguchi M, Kujuro Y, Okatsu K, Koyano F, Kosako H, Kimura M et al. Parkin-catalyzed ubiquitin-ester transfer is triggered by PINK1-dependent phosphorylation. J Biol Chem 2013; 288: 22019–22032. 23754282 10.1074/jbc.M113.467530 PMC3724655

[bib33] Kondapalli C, Kazlauskaite A, Zhang N, Woodroof HI, Campbell DG, Gourlay R et al. PINK1 is activated by mitochondrial membrane potential depolarization and stimulates Parkin E3 ligase activity by phosphorylating Serine 65. Open Biol 2012; 2: 120080. 22724072 10.1098/rsob.120080 PMC3376738

[bib34] Shiba-Fukushima K, Imai Y, Yoshida S, Ishihama Y, Kanao T, Sato S et al. PINK1-mediated phosphorylation of the Parkin ubiquitin-like domain primes mitochondrial translocation of Parkin and regulates mitophagy. Sci Rep 2012; 2: 1002. 23256036 10.1038/srep01002 PMC3525937

[bib35] Tanaka A, Cleland MM, Xu S, Narendra DP, Suen DF, Karbowski M et al. Proteasome and p97 mediate mitophagy and degradation of mitofusins induced by Parkin. J Cell Biol 2010; 191: 1367–1380. 21173115 10.1083/jcb.201007013 PMC3010068

[bib36] Chan NC, Salazar AM, Pham AH, Sweredoski MJ, Kolawa NJ, Graham RL et al. Broad activation of the ubiquitin-proteasome system by Parkin is critical for mitophagy. Hum Mol Genet 2011; 20: 1726–1737. 21296869 10.1093/hmg/ddr048 PMC3071670

[bib37] Chen Y, Dorn GW II. PINK1-phosphorylated mitofusin 2 is a Parkin receptor for culling damaged mitochondria. Science 2013; 340: 471–475. 23620051 10.1126/science.1231031 PMC3774525

