# Supplementary figures and images for: PINK1-induced mitophagy promotes neuroprotection in Huntington's disease
Source: Cell Death Dis. 2015 Jan 22;6(1):e1617–. doi: 10.1038/cddis.2014.581 (PMC4669776; doi:10.1038/cddis.2014.581)

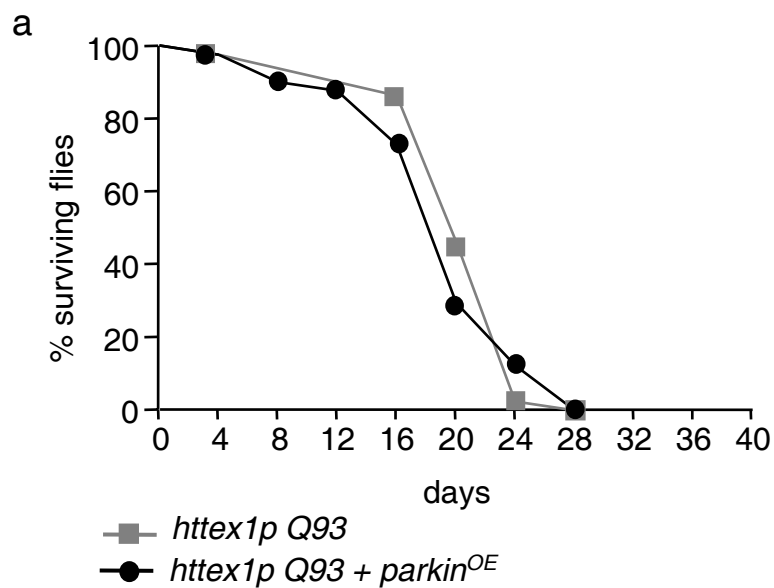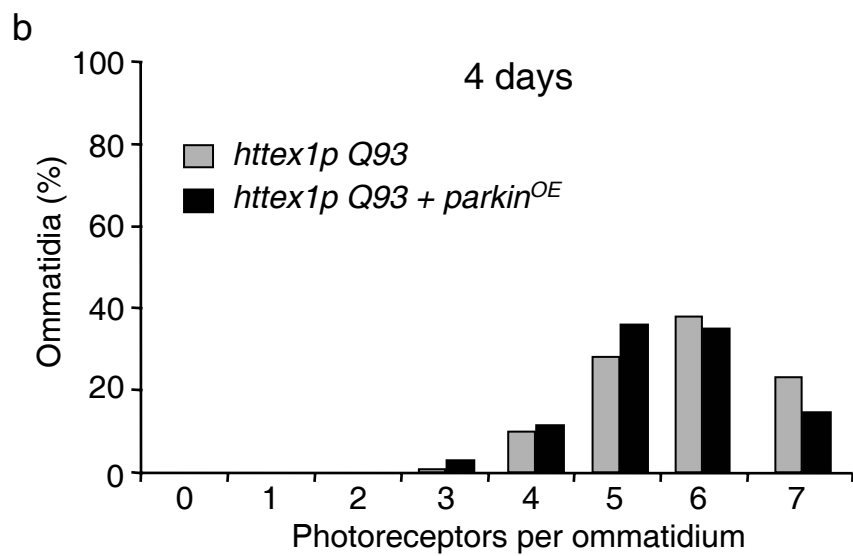

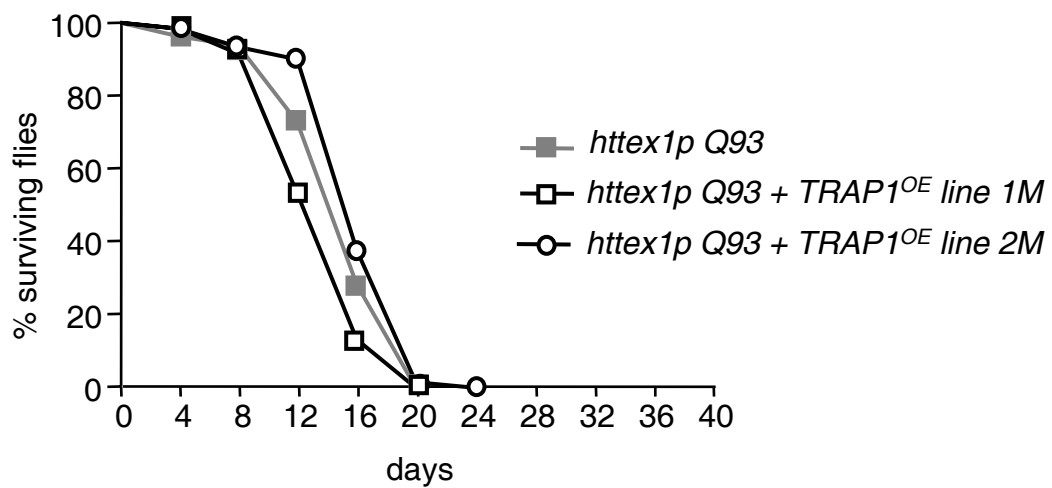

Fig. S2

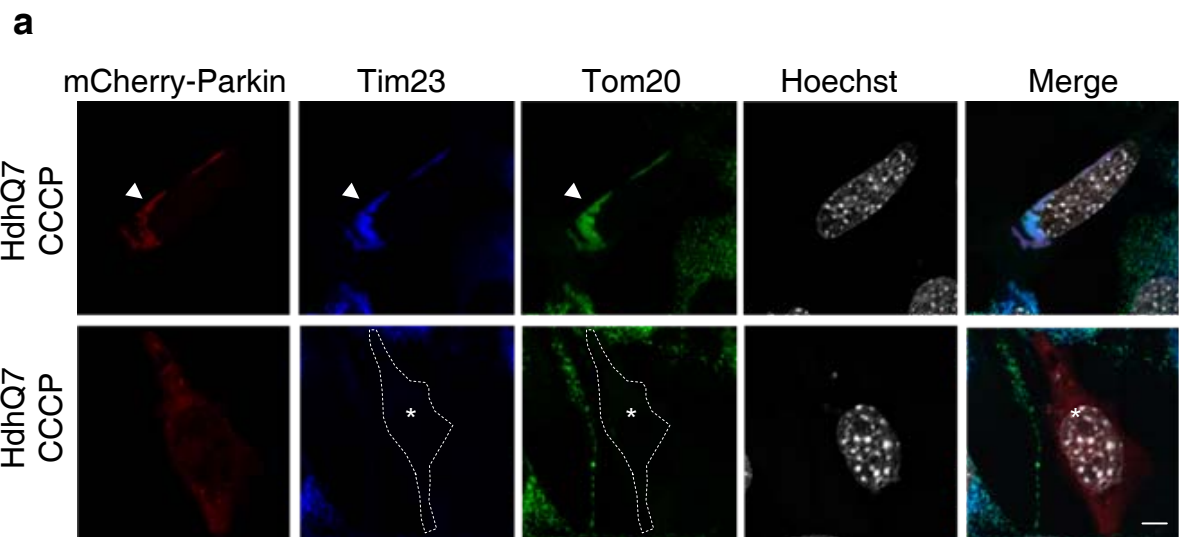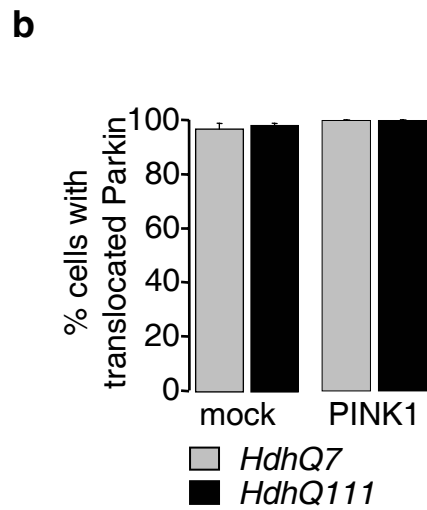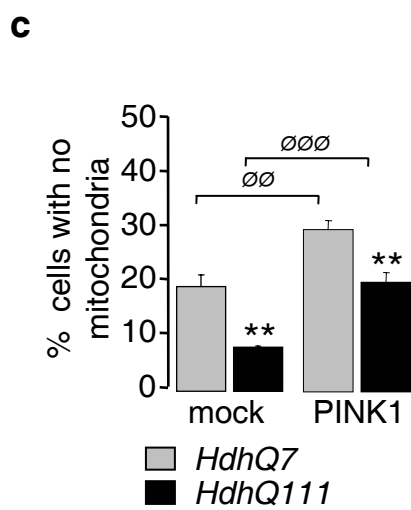

Supplement: Supplementary Figures [file cddis2014581x1.pdf]
