# Supplementary material for: PINK1-induced mitophagy promotes neuroprotection in Huntington's disease
Source: Cell Death Dis. 2015 Jan 22;6(1):e1617–. doi: 10.1038/cddis.2014.581 (PMC4669776; doi:10.1038/cddis.2014.581)
Supplement: Supplementary Figure Legends [file cddis2014581x2.doc]

**Supplemental Figures**

**Figure S1** *Drosophila* Parkin does not ameliorate neuronal death and organismal lifespan of HD flies. **(a)** Survival rates of flies expressing *httex1p Q93* alone or together with *Drosophila* *parkin* (*parkin^OE^*) under the regulation of the neuronal driver *elav-GAL4*. **(b)** Number of photoreceptors per ommatidium in 4-day old flies expressing *httex1p Q93* alone or together with *Drosophila* *parkin* (*parkin^OE^*) in neurons (n>10 flies per genotype).

**Figure S2** TRAP1 overexpression fails to mimic PINK1 neuroprotective effect on the survival of HD flies. Survival rates of flies expressing *httex1p Q93* alone, or *httex1p Q93* in the presence of *TRAP1* (*TRAP1^OE^*, lines 1M and 2M) in neurons. *TRAP1* overexpression does not modify the survival rate of HD flies (n>60 flies per genotype).

**Figure S3** PINK1 partially ameliorates deficient mitophagy in CCCP-treated HdhQ111 striatal cells. **(a)** HdhQ7 and HdhQ111 striatal cells were transfected with mCherry-Parkin together with mock plasmid or PINK1-HA and treated with 10 µM CCCP for 38 h. Mitochondria were immunostained by anti-Tim23 and anti-Tom20 antibodies and nuclei were visualized by Hoechst stain. Arrow heads show HdhQ7 cells with Parkin translocation to Tim23- and Tom20- immunostained mitochondria. White asterisk corresponds to a CCCP-treated HdhQ7 cell devoid of Tim23- and Tom20- immunostained mitochondria. Scale bar: 10 µm. **(b and c)** Quantitative analyses of the percentage of cells showing Parkin translocation to Tim23-immunostained mitochondria (a), or cells with no Tim23-immunostained mitochondria (b). Data from at least 100 cells from 3 coverslips were averaged and were presented as mean +/- SEM. Statistical analysis was performed using a Student t-test (^ØØØ^ p<0.001, ^ØØ^ p<0.01 PINK1-transfected versus mock-transfected cells; ** p<0.01 versus HdhQ7 cells).
